# Association Between HPV Vaccination and Cervical Dysplasia Severity in HPV-Positive Women

**DOI:** 10.3390/diagnostics16070979

**Published:** 2026-03-25

**Authors:** Ali Deniz Erkmen, Kevser Arkan

**Affiliations:** Division of Gynecologic Oncology, Gazi Yaşargil Training and Research Hospital, 21010 Diyarbakır, Turkey

**Keywords:** HPV vaccination, cervical dysplasia, HSIL, CIN2+, regression, adjuvant vaccination, LEEP, secondary prevention, cervical intraepithelial neoplasia, high-risk HPV

## Abstract

**Background**: Although HPV vaccination is highly effective in the primary prevention of cervical cancer, its potential role in women already diagnosed with HPV-associated cervical dysplasia remains uncertain. This study aimed to evaluate the association between post-diagnosis HPV vaccination and short-term clinical outcomes in HPV-positive women with cervical dysplasia. **Methods**: Women aged ≥18 years with abnormal cervical screening results suggestive of squamous intraepithelial lesions and high-risk HPV positivity were retrospectively evaluated. High-grade disease was defined as histologically confirmed CIN2/3. HPV vaccination (9-valent) was recommended to all eligible patients at the time of diagnosis. Vaccination status was primarily analyzed as vaccinated (≥1 dose) versus unvaccinated; additionally, dose-stratified analyses (0, 1–2, and 3 doses) were performed to explore potential dose–response relationships. **Results**: A total of 392 women were included (173 unvaccinated and 219 vaccinated). At 12 months, regression occurred in 51.1% of vaccinated patients compared with 41.0% of unvaccinated women (OR 1.50, 95% CI 1.02–2.20, *p* = 0.04). A dose–response pattern was observed, with regression rates of 41.0% in unvaccinated patients, 46.1% in partially vaccinated patients, and 54.6% in fully vaccinated patients (*p* for trend = 0.012). In the HSIL subgroup, regression occurred in 49.0% of vaccinated women versus 33.8% of unvaccinated patients (OR 1.88, 95% CI 1.01–3.52, *p* = 0.047). When stratified by treatment modality, vaccination was significantly associated with higher regression in the non-LEEP cohort (OR 1.67, *p* = 0.04) but not in the LEEP cohort (*p* = 0.22). In multivariable analysis adjusting for age, smoking, HPV genotype, baseline histopathologic grade (CIN1 vs. CIN2/3), and treatment modality, HPV vaccination remained independently associated with regression (aOR 1.55, 95% CI 1.05–2.30, *p* = 0.028). **Conclusions**: Post-diagnosis HPV vaccination was associated with a higher probability of cervical dysplasia regression at 12 months, particularly among women with baseline HSIL. These findings suggest that HPV vaccination may provide a beneficial adjunct effect in the clinical management of HPV-associated cervical dysplasia. Prospective studies are required to confirm these observations and clarify the mechanisms underlying this association.

## 1. Introduction

Persistent infection with high-risk human papillomavirus (HPV) is the principal etiologic factor underlying cervical intraepithelial neoplasia (CIN) and cervical cancer [1]. Prophylactic HPV vaccination has demonstrated robust efficacy in primary prevention, particularly when administered prior to sexual debut. Large population-based studies have confirmed significant reductions in CIN2+ incidence among vaccinated cohorts, establishing vaccination as a cornerstone of cervical cancer prevention strategies [2,3,4]. In countries with high vaccination coverage, marked declines in cervical abnormalities have been observed, raising the possibility that screening algorithms may eventually be modified in fully vaccinated populations [3,5].

Beyond primary prevention, increasing attention has been directed toward the potential role of HPV vaccination in women already diagnosed with cervical dysplasia [6]. Observational studies suggest that post-diagnosis vaccination may reduce recurrence following excisional treatment and may influence regression dynamics in low-grade lesions [7,8]. These findings have generated interest in the potential use of HPV vaccination as an adjunct in the management of established HPV-related disease.

However, interpretation of existing observational evidence remains challenging due to several methodological limitations. First, spontaneous regression, particularly in CIN1, complicates attribution of clinical improvement to vaccination rather than natural immune clearance [9,10]. Second, heterogeneity in outcome definitions, including differences between cytologic and histologic endpoints and between recurrence and regression outcomes, limits cross-study comparability [11]. Third, variability in vaccination timing and incomplete vaccination schedules introduce additional confounding, as partial vaccination may not reflect full immunologic protection [12,13]. Finally, many retrospective analyses group surgically treated and conservatively managed patients together despite fundamentally different disease trajectories following excisional treatment compared with expectant management [7,11,14].

These methodological inconsistencies make it difficult to determine whether observed reductions in recurrence or increases in regression are attributable to vaccination itself or to baseline clinical differences, spontaneous immune clearance, or treatment-related effects [15]. Consequently, whether post-diagnosis HPV vaccination independently influences short-term lesion regression—particularly in women with high-grade squamous intraepithelial lesions (HSIL), who are at increased risk of persistence or progression—remains incompletely clarified [7,16,17].

Therefore, the present retrospective cohort study aimed to evaluate the association between post-diagnosis HPV vaccination and standardized 12-month clinical outcomes in women diagnosed with LSIL or HSIL. By incorporating histology-prioritized disease classification, treatment-stratified analyses (LEEP vs. non-LEEP), dose-stratified exposure assessment, and multivariable adjustment for established clinical confounders, this study seeks to address key methodological limitations in the current literature and provide clinically relevant evidence regarding the potential role of HPV vaccination as an adjunct in the management of established cervical dysplasia.

## 2. Materials and Methods

### 2.1. Study Design and Setting

This retrospective cohort study was conducted at a tertiary gynecologic oncology center and included patients evaluated between January 2020 and December 2024. Clinical data were extracted from the hospital electronic medical record system. The study protocol was approved by Gazi Yasargil Training and Research Hospital Local Ethics Committee (date: 5 December 2025, issue: 2025/765) and was conducted in accordance with the Declaration of Helsinki. The requirement for informed consent was taken from all patients.

### 2.2. Study Population

Women aged ≥18 years who presented with abnormal cervical cytology between January 2020 and December 2024 were screened for eligibility. Cytologic findings were reported according to the Bethesda System for Reporting Cervical Cytology [18]. Patients were considered for inclusion if they had concurrent positivity for high-risk HPV DNA and subsequently underwent colposcopic evaluation according to the recommendations of the American Society for Colposcopy and Cervical Pathology (ASCCP) [19] and the European Society of Gynaecological Oncology (ESGO) [20] guidelines. Histopathologic evaluation was performed when clinically indicated through colposcopy-directed biopsy or excisional treatment (loop electrosurgical excision procedure, LEEP). For the purposes of disease classification in the present study, cervical dysplasia severity was determined primarily according to histopathologic findings when biopsy results were available. High-grade disease was therefore defined as histologically confirmed cervical intraepithelial neoplasia grade 2 or grade 3 (CIN2/3). Cytologic HSIL alone was not considered sufficient for definitive classification of high-grade dysplasia without histopathologic confirmation.

Additional inclusion criteria included absence of prior HPV vaccination and availability of complete clinical records with a minimum follow-up duration of 12 months. Exclusion criteria were previous cervical excisional or ablative treatment, pregnancy at diagnosis or during follow-up, known immunosuppressive conditions (such as HIV infection or long-term immunosuppressive therapy), and evidence of invasive cervical carcinoma at baseline evaluation. Women with cytology reported as negative for intraepithelial lesion or malignancy (NILM) were excluded to ensure that all included patients had abnormal cervical screening findings at baseline. After application of these criteria, a total of 392 patients were included in the final analysis.

### 2.3. Baseline Evaluation

At baseline, all patients underwent a standardized diagnostic workup including the following:

- Comprehensive gynecologic examination;
- Liquid-based cervical cytology;
- High-risk HPV DNA testing;
- Colposcopic examination according to guideline-based indications.

Colposcopy was performed by experienced gynecologic oncologists, and findings were documented using standardized terminology. Colposcopy-directed biopsies were obtained when indicated based on cytologic findings, colposcopic impression, or risk-based assessment algorithms. Histopathologic evaluation was performed according to established diagnostic criteria. High-risk HPV DNA testing and genotyping were performed using the Abbott RealTime High Risk HPV assay (Abbott Molecular, Des Plaines, IL, USA) on the Abbott m2000 sp/m2000 rt platform. This real-time PCR (Abbott Molecular, Des Plaines, IL, USA)-based assay detects HPV16 and HPV18 individually while identifying a pooled group of other high-risk HPV genotypes. All HPV analyses were performed in the institutional molecular diagnostics laboratory, and the same testing platform was used for all patients to ensure methodological consistency. Liquid-based cytology samples were evaluated by experienced cytopathologists in the pathology department of Gazi Yaşargil Training and Research Hospital and reported according to the Bethesda System for Reporting Cervical Cytology.

### 2.4. HPV Vaccination and Exposure Classification

Following confirmation of LSIL or HSIL, HPV vaccination (9-valent vaccine) was systematically recommended to all eligible patients at the time of diagnosis, irrespective of age, histopathologic grade (CIN1 vs. CIN2/3), HPV genotype, or management strategy. Vaccination was not reimbursed by the national healthcare system during the study period; therefore, vaccine uptake depended entirely on patient preference and financial accessibility. No sponsorship or financial support was provided by vaccine manufacturers. Vaccination status was determined through documented medical records.

HPV vaccination status was defined as the main exposure variable in this study. Patients were categorized into two groups based on vaccination history: vaccinated (receipt of at least one dose of HPV vaccine) and unvaccinated (no history of HPV vaccination). Vaccination status was determined from medical records and patient-reported vaccination history when available. For the primary analyses, patients who received one or more doses (≥1 dose) of HPV vaccine were classified as vaccinated. This approach was used to evaluate the overall association between HPV vaccination and cervical dysplasia regression. In cases where detailed vaccination information was available, the number of administered doses was recorded for descriptive purposes. This classification allowed comparison of clinical outcomes between vaccinated and unvaccinated patients while minimizing potential misclassification related to incomplete dose series or variability in vaccination schedules.

### 2.5. Clinical Management and Follow-Up

Clinical management was conducted in accordance with international guidelines, primarily those of the American Society for Colposcopy and Cervical Pathology (ASCCP) [21] and the European Society of Gynaecological Oncology (ESGO) [22]. Management decisions were individualized based on cytologic findings, HPV status, colposcopic evaluation, and histopathologic results when available. Patients with LSIL were generally managed conservatively. Follow-up evaluations typically included repeat cervical cytology and colposcopic examination at approximately 6-month intervals. Colposcopy-directed biopsies were performed when clinically indicated based on abnormal cytologic findings, suspicious colposcopic impressions, or persistence of abnormalities during follow-up. Patients with HSIL underwent a risk-based clinical evaluation incorporating cytology, HPV genotype, and colposcopic findings. When high-grade disease was suspected or confirmed, excisional treatment using loop electrosurgical excision procedure (LEEP) was performed according to standard clinical practice. All excised specimens were submitted for histopathologic examination to determine histopathologic grade and surgical margin status. In selected patients without an immediate indication for excisional treatment, careful surveillance was undertaken with repeat cytologic and colposcopic assessments. During follow-up, colposcopy-directed biopsies were obtained when clinically indicated. For outcome classification, the clinical status at the 12-month follow-up evaluation was used as the primary endpoint. When both cytologic and histopathologic results were available at the 12-month assessment, histopathologic findings were prioritized for outcome determination.

### 2.6. Outcome Definitions

The primary outcome was regression of cervical dysplasia at 12 months. Regression was defined as the absence of histopathologic evidence of cervical dysplasia or the presence of a lower-grade lesion at the 12-month follow-up evaluation. Persistence was defined as the presence of the same histopathologic grade as at baseline, whereas progression was defined as the detection of a higher-grade lesion compared with baseline findings. When both cytologic and histopathologic results were available at follow-up, histopathologic findings were prioritized for outcome classification. Regression was defined as:

- Histologic regression from CIN2/3 to CIN1 or normal epithelium;
- Histologic regression from CIN1 to normal epithelium;
- In cases without biopsy at follow-up, cytologic improvement from HSIL to LSIL or negative cytology, or from LSIL to negative cytology.

Secondary outcomes included persistence or progression of dysplasia, as well as comparative regression rates between vaccinated and unvaccinated patients within both LEEP and non-LEEP cohorts.

### 2.7. Statistical Analysis

Statistical analyses were performed using IBM SPSS Statistics software (version 25.0; IBM Corp., Armonk, NY, USA). Patients were categorized according to HPV vaccination status as the primary exposure variable. To address potential treatment-related confounding, additional analyses were stratified by treatment modality (LEEP vs. non-LEEP). Continuous variables were summarized as mean ± standard deviation or median with interquartile range, depending on distribution. Categorical variables were expressed as frequencies and percentages. Group comparisons were performed using Student’s *t*-test or Mann–Whitney U test for continuous variables and chi-square or Fisher’s exact test for categorical variables, as appropriate.

Binary logistic regression analysis was performed to evaluate the independent association between HPV vaccination and dysplasia regression. Multivariable models included clinically relevant covariates such as age, baseline histopathologic grade (CIN1 vs. CIN2/3), smoking status, HPV genotype, and treatment modality. Adjusted odds ratios (aORs) with 95% confidence intervals (CIs) were reported. A two-sided *p*-value < 0.05 was considered statistically significant.

## 3. Results

### 3.1. Baseline Clinical and Pathologic Characteristics According to Vaccination Status

Baseline demographic and clinical characteristics of the study population according to vaccination status are presented in Table 1. Vaccinated patients were significantly younger than unvaccinated women (mean age 37.9 ± 8.2 vs. 41.8 ± 9.6 years, *p* < 0.001). Parity and smoking prevalence were similar between the two groups, indicating no substantial imbalance in reproductive or behavioral characteristics. Baseline histopathologic diagnoses among patients who underwent biopsy were also comparable between vaccination groups, with similar distributions of CIN1, CIN2, and CIN3 lesions (*p* = 0.68).

The distribution of high-risk HPV genotypes—including HPV16/18, other high-risk types, and mixed infections—did not significantly differ between groups (*p* = 0.29). Excisional treatment with LEEP was more frequently performed in vaccinated patients (37.9% vs. 27.7%, *p* = 0.01). Follow-up availability at 12 months was high and comparable between cohorts (92.5% vs. 93.6%, *p* = 0.66). Overall, baseline clinical characteristics were largely balanced between groups, except for younger age and a higher rate of excisional treatment among vaccinated patients.

Baseline colposcopic findings and histopathologic biopsy results according to vaccination status are shown in Table 2. Baseline colposcopic evaluation was performed in nearly all patients in both vaccination groups, with no significant difference in the proportion of women undergoing colposcopy (96.0% vs. 97.3%, *p* = 0.42). The distribution of transformation zone types was similar between groups, with TZ2 representing the most common configuration in both cohorts.

Colposcopic impressions demonstrated comparable distributions of normal, low-grade, and high-grade patterns (*p* = 0.31). Baseline colposcopy-directed biopsies were obtained in 75.7% of unvaccinated and 78.5% of vaccinated patients (*p* = 0.51). Among biopsied patients, histopathologic diagnoses, including negative findings, CIN1, CIN2, CIN3, and adenocarcinoma in situ (AIS), were distributed similarly across the two groups (*p* = 0.68). These findings indicate that baseline colposcopic and histopathologic disease severity was comparable between vaccinated and unvaccinated patients.

Histopathologic characteristics of LEEP specimens and surgical margin status according to vaccination status are presented in Table 3. Among patients who underwent excisional treatment, the median time from diagnosis to LEEP was similar between vaccinated and unvaccinated women (median 4 weeks in both groups, *p* = 0.88). Histopathologic examination of LEEP specimens demonstrated comparable distributions of CIN grades across groups. CIN3 represented the most frequent diagnosis in both cohorts, followed by CIN2.

Margin status was also similar between groups. The rates of positive endocervical and ectocervical margins did not significantly differ between vaccinated and unvaccinated patients (*p* = 0.84 and *p* = 0.91, respectively). Likewise, the frequencies of glandular involvement and endocervical curettage (ECC) positivity were comparable. Overall, excisional pathology findings were balanced between cohorts, suggesting no major differences in surgical specimen characteristics that could influence recurrence risk. All patients were followed for a minimum of 12 months after diagnosis, ensuring comparable follow-up duration across both LEEP and non-LEEP cohorts.

### 3.2. Follow-Up Evaluation and Outcome Assessment at 12 Months

Follow-up assessment methods and endpoint classification at 12 months are summarized in Table 4. Outcome data at 12 months were available for most patients in both treatment groups, with no significant differences in follow-up completeness across vaccination strata (*p* = 0.73). In the non-LEEP cohort, outcome classification was generally based on cytologic assessment during routine surveillance, whereas histologic confirmation was more frequently available among patients who underwent excisional treatment.

However, the overall distribution of endpoint assessment methods (cytology-only versus histology-available) did not significantly differ between vaccination groups (*p* = 0.48). Similarly, the proportions of patients undergoing follow-up colposcopy or colposcopy-directed biopsy were comparable between vaccinated and unvaccinated cohorts (*p* = 0.29 and *p* = 0.41, respectively). These findings suggest that outcome ascertainment and surveillance intensity were similar across groups and were unlikely to introduce systematic measurement bias.

Twelve-month dysplasia outcomes according to the number of vaccine doses received are presented in Table 5. When regression rates were analyzed according to the number of vaccine doses received after diagnosis, a dose-dependent trend was observed. Regression occurred in 41.0% of unvaccinated patients, 46.1% of partially vaccinated patients, and 54.6% of fully vaccinated patients. Compared with unvaccinated women, partially vaccinated patients demonstrated a modest but statistically non-significant increase in regression (OR 1.23, *p* = 0.40), whereas fully vaccinated patients had significantly higher odds of regression (OR 1.72, 95% CI 1.12–2.63, *p* = 0.013). A trend analysis across the three exposure categories demonstrated a significant dose–response relationship (*p* for trend = 0.012).

### 3.3. Association Between HPV Vaccination and Dysplasia Regression

HPV vaccination was associated with a higher probability of cervical dysplasia regression at 12 months. In the overall cohort, regression occurred in 112 of 219 vaccinated patients (51.1%) compared with 71 of 173 unvaccinated patients (41.0%), corresponding to an odds ratio (OR) of 1.50 (95% CI: 1.02–2.20; *p* = 0.04). Among patients with baseline HSIL, regression was observed in 49.0% of vaccinated patients compared with 33.8% of unvaccinated patients (OR 1.88, 95% CI: 1.01–3.52; *p* = 0.047). When stratified by treatment modality, vaccination was not significantly associated with regression in the LEEP cohort (OR 1.60, 95% CI: 0.75–3.40; *p* = 0.22), whereas a significant association was observed in the non-LEEP cohort (OR 1.67, 95% CI: 1.02–2.73; *p* = 0.04). These findings indicate that HPV vaccination was associated with increased regression rates, particularly among patients managed conservatively. The full results of the univariate analyses are presented in Table 6.

### 3.4. Regression Outcomes Stratified by Treatment Modality (LEEP vs. Non-LEEP)

Dysplasia outcomes stratified by treatment modality and vaccination status are presented in Table 7. When outcomes were stratified according to treatment modality, regression remained consistently more frequent in vaccinated patients across both LEEP and non-LEEP cohorts. In the LEEP cohort, regression was observed in 39.8% of vaccinated patients compared with 29.2% of unvaccinated women. In the non-LEEP cohort, regression occurred in 58.1% of vaccinated patients and 45.6% of unvaccinated patients. Although the magnitude of effect varied between treatment strata, the direction of association remained consistent, supporting a potential adjuvant role of vaccination regardless of management strategy. Further detailed regression patterns in patients with baseline HSIL was provided in Supplementary Table S1.

### 3.5. Multivariable Logistic Regression Analysis of Factors Associated with Dysplasia Regression

Multivariable logistic regression analysis was performed to identify independent predictors of cervical dysplasia regression at 12 months (Table 8). After adjustment for age, smoking status, high-risk HPV genotype, treatment modality, and baseline histopathological grade, HPV vaccination remained independently associated with an increased likelihood of regression (adjusted OR 1.74, 95% CI 1.12–2.69, *p* = 0.014). Baseline histopathologic severity also showed a significant association with regression outcomes. Patients with CIN2/3 at baseline had a significantly lower probability of regression compared with those with CIN1 (adjusted OR 0.58, 95% CI 0.38–0.89, *p* = 0.012). Age, smoking status, HPV genotype, and treatment modality were not independently associated with regression in the adjusted model.

## 4. Discussion

In this retrospective cohort study, we evaluated the association between post-diagnosis HPV vaccination and the 12-month clinical evolution of cervical dysplasia. Our findings suggest that HPV vaccination administered after diagnosis is associated with higher rates of lesion regression, particularly among women presenting with HSIL. The magnitude and consistency of this association across overall and stratified analyses support the hypothesis that vaccination may exert an adjuvant effect in patients with established high-risk cervical dysplasia. An additional strength of the present study is the dose-stratified evaluation of vaccination exposure. When patients were categorized according to the number of vaccine doses received after diagnosis, a gradient in regression rates was observed. Fully vaccinated women demonstrated the most pronounced regression patterns, whereas partially vaccinated patients exhibited intermediate outcomes. Although the statistical significance of partial vaccination was not consistent across all analyses, this pattern suggests a potential dose–response relationship that supports the biological plausibility of the observed association.

The relationship between HPV vaccination and dysplasia severity must be interpreted within the broader context of primary versus secondary prevention [23]. In fully vaccinated prophylactic cohorts, a substantial reduction in high-grade lesions (CIN2+) is well established [10]. For example, Valent et al. [24] reported a marked reduction in CIN2+ prevalence among vaccinated women compared with unvaccinated controls (0.2% vs. 3.6%). However, unadjusted observational analyses may produce counterintuitive findings. In Scottish surveillance data, Pollock et al. [25] observed an apparently increased relative risk of CIN3 among women who had received only one vaccine dose in crude analyses (RR 1.89), a finding that disappeared after adjustment for deprivation and cohort effects. These results illustrate how incomplete vaccination may act as a proxy for socioeconomic or behavioral risk factors rather than reflect vaccine inefficacy. In our cohort, vaccinated patients were younger and more frequently underwent excisional treatment, suggesting potential clinical selection patterns. This phenomenon is consistent with what has been described as “confounding by indication” in retrospective adjuvant vaccination studies. Health technology assessments, including the review by van de Laar [26], have emphasized that in non-randomized prospective and retrospective settings, women who elect to receive vaccination may differ systematically in health literacy, socioeconomic status, or healthcare engagement. Similarly, Ghelardi et al. [27] highlighted the risk of self-selection bias in post-treatment vaccination cohorts.

Age-related confounding is particularly relevant. Petrillo et al. [28] reported that vaccinated women in their post-LEEP cohort were significantly younger than unvaccinated controls, a difference that may independently influence immune response and spontaneous regression rates. In the context of low-grade lesions, Gardella et al. [29] demonstrated that age and viral genotype were strong predictors of lesion evolution, and that rigorous multivariable adjustment was required to isolate the independent contribution of vaccination. In our study, multivariable analysis adjusting for age, baseline histopathological grade, smoking status, HPV genotype, and treatment modality demonstrated that vaccination remained independently associated with regression, suggesting that the observed association is not fully explained by demographic imbalance.

The attenuation of crude associations after adjustment has been a recurring theme in HPV vaccination research [30]. In the Scottish cohort, the elevated crude risk of CIN3 in partially vaccinated women became non-significant after adjustment for socioeconomic deprivation and calendar year [25]. Likewise, in the New Mexico population-based data, Otto [31] emphasized that failure to account for changes in screening intervals could lead to overestimation of vaccine impact. These studies underscore the importance of multivariable modeling when interpreting observational data. Our adjusted model demonstrated that vaccination was independently associated with increased odds of regression, whereas baseline HSIL and smoking were associated with reduced regression probability—findings that are biologically and clinically coherent.

The effect observed in our HSIL subgroup is important. Regression rates among vaccinated HSIL patients were substantially higher than among unvaccinated women. This aligns with the broader evidence suggesting that the magnitude of benefit may be more apparent in higher-risk subgroups. In a meta-analysis of post-conization cohorts, Maiorano et al. [7] reported a 62% relative reduction in CIN2+ recurrence among vaccinated women. However, the underlying mechanism remains debated. Maiorano et al. [7] and Petrillo et al. [28] argue that the benefit in surgically treated patients is likely prophylactic, preventing reinfection or reactivation in susceptible tissue rather than directly clearing persistent viral infection. In contrast, Gardella et al. [29] suggested that vaccination may enhance immune-mediated clearance of existing low-grade lesions. Our findings, particularly in conservatively managed HSIL patients, are compatible with the hypothesis that immune augmentation may influence the short-term natural history of dysplasia, although causal inference cannot be established. Treatment modality represents another important factor influencing clinical outcomes in cervical dysplasia. Excisional procedures such as LEEP can substantially alter the natural course of HPV-related lesions by physically removing dysplastic tissue. To address this potential source of heterogeneity, we performed stratified analyses according to treatment modality. The association between vaccination and lesion regression remained consistent across treatment strata, suggesting that the observed findings are unlikely to be explained solely by treatment-related effects.

Stratification by treatment modality provided additional insight. Regression rates were higher among vaccinated women in both LEEP and non-LEEP cohorts, with a somewhat stronger association observed in conservatively managed patients. This pattern may reflect the interplay between natural regression and immune modulation in intact cervical epithelium. In the LEEP cohort, the relative contribution of vaccination may be less pronounced in the short term, as excisional treatment itself removes dysplastic tissue, thereby modifying measurable disease dynamics.

Several limitations must be acknowledged. The retrospective design precludes definitive causal inference, and residual confounding cannot be excluded despite multivariable adjustment. Additionally, detailed sexual behavior variables such as number of lifetime partners or age at sexual debut were not systematically recorded in the electronic medical records and therefore could not be included in the analysis. These factors may influence HPV persistence and dysplasia outcomes and represent a potential source of residual confounding. Vaccination uptake was not randomized and may reflect unmeasured behavioral or socioeconomic factors. Additionally, while histopathology was prioritized for outcome classification, some assessments relied on cytology, which may introduce minor misclassification. Finally, follow-up was limited to 12 months; longer-term outcomes, including persistent high-risk HPV infection and late recurrence, were not evaluated.

## 5. Conclusions

In this retrospective cohort study, HPV vaccination administered after the diagnosis of cervical dysplasia was associated with higher regression rates at 12 months. The association remained significant after adjustment for key clinical variables and was most pronounced among patients with baseline HSIL. A dose–response trend was also observed, suggesting a potential incremental benefit with complete vaccination. Although these findings support the hypothesis that HPV vaccination may provide an adjunct benefit in women with established cervical dysplasia, the retrospective design and the lack of data on socio-cultural and sexual practices in the study population limit causal inference. Residual confounding and other unmeasured behavioral factors cannot be fully excluded. Larger prospective and randomized studies with longer follow-up and standardized virological assessment are needed to confirm these results and clarify the underlying biological mechanisms. Importantly, HPV vaccination should not be considered a therapeutic intervention for established cervical dysplasia. Vaccination may serve as a potential adjunct strategy but does not replace standard clinical management. Regular cervical cancer screening and guideline-based follow-up remain essential for all eligible women, regardless of vaccination status.

## Figures and Tables

**Table 1:** Baseline demographic and histopathologic characteristics of the study population according to HPV vaccination status.

Variable		Unvaccinated (0 Dose) (*n* = 173)	Vaccinated (≥1 Dose) (*n* = 219)	*p*-Value
Age, years (mean ± SD)		41.8 ± 9.6	37.9 ± 8.2	<0.001
Parity, median (IQR)		3 (2–4)	3 (2–4)	0.44
Current smoker, *n* (%)		64 (37.0%)	78 (35.6%)	0.78
Baseline histopathology (when biopsy performed), *n* (%) *	Negative	13 (9.9%)	16 (9.3%)	0.68
	CIN1	46 (35.1%)	58 (33.7%)	
	CIN2	34 (26.0%)	45 (26.2%)	
	CIN3	36 (27.5%)	49 (28.5%)	
	AIS	2 (1.5%)	4 (2.3%)	
High-risk HPV genotype, *n* (%)	HPV 16/18	71 (41.0%)	82 (37.4%)	0.29
	Other HR-HPV	86 (49.7%)	116 (53.0%)	
	Mixed infection (16/18 + other)	9 (5.2%)	12 (5.5%)	
	Unknown/Not genotyped	7 (4.0%)	9 (4.1%)	
Treatment modality during follow-up, *n* (%)	LEEP performed	48 (27.7%)	83 (37.9%)	0.01
	Conservative management	125 (72.3%)	136 (62.1%)	
12-month outcome available, *n* (%)		160 (92.5%)	205 (93.6%)	0.66

Continuous variables are presented as mean ± standard deviation (SD) or median with interquartile range (IQR), depending on distribution. Categorical variables are expressed as number (percentage). Between-group comparisons were performed using Student’s *t*-test or Mann–Whitney U test for continuous variables and the chi-square test or Fisher’s exact test for categorical variables, as appropriate. HPV genotype classification was based on high-risk categories, with HPV16 and HPV18 considered the highest oncogenic risk types. * Baseline disease status was determined according to histopathologic findings from colposcopy-directed biopsies when available.

**Table 2:** Baseline colposcopic findings and histopathologic biopsy results according to HPV vaccination status.

Variable		Unvaccinated (*n* = 173)	Vaccinated (*n* = 219)	*p*-Value
Colposcopy performed, *n* (%)		166 (96.0%)	213 (97.3%)	0.42
Transformation zone type	TZ1	55 (33.1%)	72 (33.8%)	0.94
	TZ2	77 (46.4%)	97 (45.5%)	
	TZ3	34 (20.5%)	44 (20.7%)	
Colposcopic impression	Normal/benign	23 (13.9%)	27 (12.7%)	0.31
	Low-grade	74 (44.6%)	88 (41.3%)	
	High-grade	65 (39.2%)	93 (43.7%)	
	Unsatisfactory	4 (2.4%)	5 (2.3%)	
Biopsy taken at baseline		131 (75.7%)	172 (78.5%)	0.51
Baseline biopsy histology	Negative	13 (9.9%)	16 (9.3%)	0.68
	CIN1	46 (35.1%)	58 (33.7%)	
	CIN2	34 (26.0%)	45 (26.2%)	
	CIN3	36 (27.5%)	49 (28.5%)	
	AIS	2 (1.5%)	4 (2.3%)	

TZ: Transformation zone. CIN: Cervical Intraepithelial Neoplasia Categorical variables are expressed as number (percentage). Comparisons between groups were performed using the chi-square test or Fisher’s exact test, as appropriate.

**Table 3:** Histopathologic characteristics and surgical margin status of LEEP specimens according to vaccination status.

Variable		Unvaccinated + LEEP (*n* = 48)	Vaccinated + LEEP (*n* = 83)	*p*-Value
Time to LEEP (weeks), median (IQR)		4 (3–6)	4 (3–6)	0.88
LEEP specimen histology	≤CIN1	5 (10.4%)	7 (8.4%)	0.77
	CIN2	16 (33.3%)	26 (31.3%)	
	CIN3	26 (54.2%)	48 (57.8%)	
	AIS	1 (2.1%)	2 (2.4%)	
Endocervical margin	Negative	34 (70.8%)	58 (69.9%)	0.84
	Positive	12 (25.0%)	22 (26.5%)	
	Indeterminate	2 (4.2%)	3 (3.6%)	
Ectocervical margin	Negative	38 (79.2%)	66 (79.5%)	0.91
	Positive	8 (16.7%)	14 (16.9%)	
	Indeterminate	2 (4.2%)	3 (3.6%)	
Glandular involvement		6 (12.5%)	11 (13.3%)	0.89
ECC performed		35 (72.9%)	62 (74.7%)	0.81

LEEP: loop electrosurgical excision procedure. ECC: Endo-Cervical Curettage. Between-group comparisons were performed using the Mann–Whitney U test for continuous variables and the chi-square or Fisher’s exact test for categorical variables, as appropriate.

**Table 4:** Follow-up assessment methods and endpoint classification at 12 months according to treatment cohort.

Variable		Non-LEEP Unvacc (*n* = 125)	Non-LEEP Vacc (*n* = 136)	LEEP Unvacc (*n* = 48)	LEEP Vacc (*n* = 83)	*p*-Value
12-month available		116 (92.8%)	128 (94.1%)	44 (91.7%)	77 (92.8%)	0.73
Endpoint type (overall comparison)	Cytology-only	98	107	12	20	0.48
	Histology-available	18	21	32	57	
Follow-up colposcopy		36 (28.8%)	43 (31.6%)	27 (56.3%)	50 (60.2%)	0.29
Follow-up biopsy		19 (15.2%)	22 (16.2%)	14 (29.2%)	26 (31.3%)	0.41

Comparisons between vaccination groups were performed using the chi-square or Fisher’s exact test, as appropriate. Percentages represent proportions within each treatment cohort.

**Table 5:** Twelve-month dysplasia outcomes according to number of HPV vaccine doses received after diagnosis.

Vaccination Status	*n*	Regression *n* (%)	Persistence *n* (%)	Progression *n* (%)	OR (95% CI)	*p*-Value
0 dose	173	71 (41.0%)	88 (50.9%)	14 (8.1%)	Reference	—
1–2 doses	89	41 (46.1%)	41 (46.1%)	7 (7.9%)	1.23 (0.75–2.02)	0.40
3 doses	130	71 (54.6%)	53 (40.8%)	6 (4.6%)	1.72 (1.12–2.63)	0.013

Regression was defined as histologic regression from CIN2/3 to CIN1 or normal epithelium, or from CIN1 to normal epithelium. In patients without biopsy at follow-up, regression was defined as cytologic improvement from HSIL to LSIL or negative cytology, or from LSIL to negative cytology. Odds ratios (ORs) with 95% confidence intervals (CIs) represent crude estimates derived from unadjusted logistic regression models comparing each vaccination group with the unvaccinated reference group. Trend analysis across vaccination dose categories was performed using a Cochran–Armitage trend test.

**Table 6:** Association between HPV vaccination and cervical dysplasia regression at 12 months in the overall cohort and selected subgroups.

Cohort	Outcome	Unvaccinated *n/N* (%)	Vaccinated *n/N* (%)	OR (95% CI)	*p*-Value
Overall cohort	Regression	71/173 (41.0%)	112/219 (51.1%)	1.50 (1.02–2.20)	0.04
	Persistence	88/173 (50.9%)	94/219 (42.9%)	–	–
	Progression	14/173 (8.1%)	13/219 (5.9%)	–	–
Baseline HSIL subgroup	Regression	22/65 (33.8%)	47/96 (49.0%)	1.88 (1.01–3.52)	0.047
	Persistence	35/65 (53.8%)	42/96 (43.8%)	–	–
	Progression	8/65 (12.3%)	7/96 (7.3%)	–	–
LEEP cohort	Regression	14/48 (29.2%)	33/83 (39.8%)	1.60 (0.75–3.40)	0.22
	No regression	34/48 (70.8%)	50/83 (60.2%)	–	–
Non-LEEP cohort	Regression	57/125 (45.6%)	79/136 (58.1%)	1.67 (1.02–2.73)	0.04
	No regression	68/125 (54.4%)	57/136 (41.9%)	–	–

ORs were calculated using univariate logistic regression comparing vaccinated and unvaccinated patients. The outcome variable was defined as regression versus no regression (persistence or progression).

**Table 7:** Twelve-month dysplasia outcomes stratified by vaccination status and treatment modality.

Cohort	*n*	Regression *n* (%)	Persistence *n* (%)	Progression *n* (%)
Vaccinated + LEEP	83	33 (39.8%)	44 (53.0%)	6 (7.2%)
Unvaccinated + LEEP	48	14 (29.2%)	29 (60.4%)	5 (10.4%)
Vaccinated + Non-LEEP	136	79 (58.1%)	53 (39.0%)	4 (2.9%)
Unvaccinated + Non-LEEP	125	57 (45.6%)	61 (48.8%)	7 (5.6%)

Patients were stratified according to treatment modality during follow-up (LEEP vs. conservative management). Regression, persistence, and progression were defined according to standardized 12-month outcome classification. Percentages represent proportions within each treatment stratum.

**Table 8:** Multivariable logistic regression analysis of factors associated with regression of cervical dysplasia at 12 months.

Predictor	aOR	95% CI	*p*-Value
Vaccinated (≥1 dose vs. 0)	1.55	1.05–2.30	0.028
Age (per year)	0.99	0.97–1.01	0.40
Baseline histopathologic grade *	0.58	0.38–0.89	0.012
Smoking (yes vs. no)	0.70	0.48–1.02	0.063
HPV16/18 (vs. other)	0.68	0.46–1.00	0.051
LEEP (yes vs. no)	0.85	0.56–1.30	0.46

Multivariable logistic regression analysis was performed using a forced-entry model including age, baseline hstopathologic grade, smoking status, HPV genotype, and treatment modality as covariates. Adjusted odds ratios (aORs) are presented with 95% confidence intervals (CIs). Statistical significance was defined as a two-sided *p*-value < 0.05. * Baseline histopathologic grade was modeled as CIN2/3 versus CIN1 among patients with available baseline biopsy-confirmed dysplasia.

## Data Availability

The data generated and analyzed during this study are presented in this manuscript.

## Supplementary Materials

The following supporting information can be downloaded at: https://www.mdpi.com/article/10.3390/diagnostics16070979/s1, Table S1. Detailed regression patterns in patients with baseline HSIL.
