# Supplementary material for: Association Between HPV Vaccination and Cervical Dysplasia Severity in HPV-Positive Women
Source: Diagnostics (Basel). 2026 Mar 25;16(7):979. doi: 10.3390/diagnostics16070979 (PMC13073047; doi:10.3390/diagnostics16070979)
Supplement: Supplementary file 1 [file diagnostics-16-00979-s001.zip › diagnostics-4180833-supplementary.pdf]

Supplementary Tables

When regression patterns were further analyzed, complete regression to negative cytology was observed more frequently among vaccinated patients compared with unvaccinated women, whereas regression to LSIL showed similar proportions between groups (Supplementary Table S1).

Supplementary Table S1. Detailed regression patterns in patients with baseline HSIL

**Outcome (baseline HSIL)**	**Unvaccinated (n=65)**	**Vaccinated (n=96)**	**p-value**
HSIL → Negative	8 (12.3%)	22 (22.9%)	0.04
HSIL → LSIL	14 (21.5%)	25 (26.0%)	0.51
Persistence	35 (53.8%)	42 (43.8%)	0.21
Progression	8 (12.3%)	7 (7.3%)	0.28
